# *In ovo* nano-silver and nutrient supplementation improves immunity and resistance against Newcastle disease virus challenge in broiler chickens

**DOI:** 10.3389/fvets.2022.948069

**Published:** 2022-09-16

**Authors:** Subrat Kumar Bhanja, Pradeepta Kumar Rath, Akshat Goel, Manish Mehra, Sujoy K. Dhara, Vinod K. Paswan, Youssef A. Attia, Abdulmohsen Hussen Alqhtani, Ahmed B. A. Ali, Abdelrazeq M. Shehata

**Affiliations:** ^1^ICAR-Central Avian Research Institute, Bareilly, UP, India; ^2^ICAR-Indian Veterinary Research Institute, Bareilly, UP, India; ^3^Department of Dairy Science and Food Technology, Institute of Agricultural Sciences, Banaras Hindu University, Varanasi, India; ^4^Department of Animal and Poultry Production, Faculty of Agriculture, Damanhour University, Damanhour, Egypt; ^5^Animal Production Department, Food and Agriculture Sciences College, King Saud University, Riyadh, Saudi Arabia; ^6^Department of Animal and Veterinary Science, Clemson University, Clemson, SC, United States; ^7^Department of Animal Production, Faculty of Agriculture, Al-Azhar University, Cairo, Egypt

**Keywords:** silver nanoparticles, *in ovo* feeding, amino acids, vitamins and minerals, immunity, ND virus challenge, gene expression, broiler chickens

## Abstract

Silver nanoparticles (AgNPs) interact with the microbes and host immune system to protect against diseases. Fertile broiler eggs (*n* = 900) were allotted to six groups: un-injected control, sham (sterile water), AgNPs (50 μg), AgNPs+Amino acids (Methionine-10 mg + Arginine-25 mg), AgNPs+Vitamins (Vit B1-72μg + Vit B6-140μg), and AgNPs+Trace Elements (Zn-80 μg and Se-0.3 μg) and incubated for 18 days. On 18th embryonic day, 0.6 ml test solution was injected at the broad end of egg using 25 mm needle and transferred to hatcher. Post-hatch, half of the chicks from each group were vaccinated with Newcastle disease (ND) vaccine, and the other half were kept as unvaccinated unit and reared for 42 d with standard management practices. Hatchability, 1st and 42nd d body weight, feed intake, and feed conversion ratio were similar between treatment groups in both vaccinated and unvaccinated units. The relative weight of bursa Fabricius and thymus was similar, but spleen weight was higher (*P* ≤ 0.05) in AgNPs, AgNPs+Vits, and AgNPs+TEs chicks than control group. Cellular immune response (against mitogen phytohemagglutinin-P) was higher (*P* ≤ 0.05) in AgNPs+TEs chicks, whereas HA titer against sheep red blood cells antigen, serum IgG, IgM, and HI titer against ND vaccine was apparently higher in AgNPs+Vits group chicks than control. No clinical symptoms were observed in the vaccinated groups except for a few control birds 6 days postchallenge (PC). Three days PC, unvaccinated birds show depression, off feed, greenish diarrhea, and nasal discharge and the control group started dying. The highest cumulative infection (CI) was observed in sham (79.17%) and un-injected control (75%), but lowest in AgNPs+AAs birds (58.33%) on 3rd dpi. The CI reached 100% on 5th dpi in control groups and AgNPs, and 91.67% and 93.75% in AgNPs+TEs and AgNPs+AAs group, respectively. The AgNPs+TEs and AgNPs+AAs group birds lived for more than 90 h compared to 75 h in control groups and also had higher IL-6 and IL-2 gene expressions at 24 h PC. It was concluded that 50 μg/egg AgNPs with vitamins (B1 and B6) and trace elements (Zn and Se) improved performance, but AgNPs with trace elements and amino acids enhanced immune response and resistance against vND virus challenge in broilers.

## Introduction

Modern broilers have tremendous improvement in the growth performance, but exhibit weaker immunity (1, 2). Loss of immune homeostasis is attributed to heavy vaccinations, early medications, transport stress, housing environment, climatic conditions, nutrition, and diseases (3–7). Thus, to achieve higher and effective poultry production, a balance between nutrition and immune status of broiler chickens would play a very significant role.

The nanomaterials have become the contemporary area of interest, for their application in the fields of animal nutrition, health, production, and many other such factors. Due to their higher surface area-to-volume ratio, these particles act better than their bulky counterparts (8–11). Recent studies reported that administration of silver nanoparticles (AgNPs) improves late embryonic development and metabolism (12–15), influencing the expression of VEGFA and FGF2 gene in the breast muscle of broilers (16), immune response, and enhancing expression of immune-related genes (17). However, the impacts of intervention with nanoparticles are yet to be fully assessed on post-hatch growth, nutrient–gene interaction, immune-competence, and disease resistance in chicken. Moreover, silver nanoparticles interact with virus, bacteria, and immune system as well. Development of B and T lymphocytes initiates during embryogenesis in the bursa of Fabricius and thymus, respectively, and matures in the spleen until post-hatch (18). The cell produced in these organs differentiates into Th1 type (cellular immunity) and Th2 type (humoral immunity) and thus imparting immunity against the different pathogens. Goel et al. (17) reported that *in ovo* injection of AgNPs enhanced the *in vivo* immune response to phytohemagglutinin type-P (PHA-P**)**, the cell-mediated immunity and sheep red blood cells (SRBC), and the humoral immunity in comparison with control group. Saki and Salary (19) have also reported about the enhanced cell-mediated immunity in terms of mean skin thickness sensitive to phytohemagglutinin, in chicks treated with AgNPS. Similarly, the application of 1% AgNP cream (<50 nm particle size) in rats inhibited contact allergic dermatitis and modulated cytokine excretion *in vitro* and *in vivo* (20). AgNP administration (0.5 mg/Kg body weight) had no significant effect on growth performance but showed a lower plasma cholesterol, triglycerides, and antioxidant capacity but better malondialdehyde and glutathione peroxidase than that of control rabbits (21).

Early post-hatch or embryonic (*In ovo)* feeding has been helpful in improving the nutritional status of the hatchlings. During the early period of embryonic growth, there is higher utilization of amino acids like Lys, Arg, Gly, and Pro. *In ovo* fed amino acids also spare the maternal antibodies from being utilized as protein source. Earlier studies revealed that *in ovo* feeding of Met and Arg had higher percent hatchability, body weight, and cell-mediated immune response (22, 23). *In ovo* administration of combined amino acids Lys+Met+Cys and Thr+Gly+Ser improved both cellular and humoral immunity (24). Thiamin (vitamin B1) helps in carbohydrate metabolism and is required for growth, development, and function of cells. Pyridoxine (vitamin B6) plays an important role in protein synthesis needed for an immune response. *In ovo* feeding of vitamin B1 improved growth and immunity, while vitamin B6 modulates immunity in broiler chickens (25). Zinc (Zn) and selenium (Se) play a vital role in various biological processes as a component of many enzymes and are also essential for growth, antioxidant defense mechanism (26), cell-mediated immunity, and higher expression of growth and immune genes (27–29). AgNPs have mainly been studied for their antimicrobial potential against bacteria, but have also proven to be active against several types of viruses, including human immunodeficiency virus, hepatitis B virus, herpes simplex virus, respiratory syncytial virus, and monkey pox virus (30–32). Silver's mode of action is presumed to be dependent on its positive ions, which strongly inhibit bacterial growth through suppression of respiratory enzymes and electron transport components and interference with DNA functions (33). Silver has also been found to be non-toxic to humans at very small concentrations. The microorganisms are unlikely to develop resistance against Ag as compared to antibiotics, as Ag attacks a broad range of targets in the microbes. The overuse of antibiotics in the standard management practices of modern broiler production systems has resulted in an increased number of antibiotic-resistant bacteria, posing imminent threat to human health. Hence, alternative health-promoting agents are looked for enhancing growth and immunity in chickens (10, 11, 34).

Though AgNPs are used in various fields, until now very few studies have been undertaken to determine the immunological effects of AgNPs when delivered to broiler chickens especially before hatch *via in ovo* feeding. Therefore, this study was planned to demonstrate how AgNPs alone or in a combination with other critical nutrients can act as an inducer of innate or adaptive immunity in broiler chickens.

## Materials and methods

### Ethical permission

All the experimental procedures on birds were carried out according to the recommendations and approval of the ICAR-Central Avian Research Institute, Izatnagar, India's Institute Animal Ethics Committee vides approval no. CARI/CPCSEA/2016/8 dated 23.08.2016 for the Purpose of Control and Supervision of Experiments on Animals in India.

### Experimental design

In a completely randomized block design, 900 fertile eggs (58.0 ± 2.0 g) collected from 35th-week-old breeder hens in a 4-day period were allotted to six groups. The eggs were incubated in a force draft incubator at standard incubation temperature of 37.5^0^C and relative humidity of 60.0 % for 18 d.

### Silver nanoparticles

The AgNPs solution aXonnite (100 mg/l deionized water, 99.9999% purity) was obtained from Nano-Tech Poland Ltd (Warsaw, Poland). The average particle size was 3.5 nm, which accounts for more than 80% of particles. The average surface area of the particles was 2.83 10^−13^ cm^2^. The pH of the solution was in the range of 7.1 to 8.1, and the redox potential was 0.1 mV.

### Treatment groups and *in ovo* injection

The six treatment groups were as follows: 1-Un-injected control, 2-Sham control (Sterile water), 3-AgNPs (50 μg), 4-AgNPs+Amino acids: Methionine-10 mg and Arginine 25 mg (AgNPs+AAs), 5-AgNPs+Vitamins: Thiamine-72 μg and Pyridoxine-140 μg (AgNPs+Vits) and 6-AgNPs+Trace elements: Zinc 80 μg and Selenium 0.3 μg (AgNPs+TEs). The concentration of critical nutrients like amino acids, trace elements, and vitamins was calculated at 5, 50, and 100%, respectively, of the National Research Council (35) recommendation for a broiler chick's consumption during first 4 d (22). Separate nutrient solutions were prepared by dissolving amino acids, vitamins, and trace elements in sterile water, and then, these nutrient solutions (0.1 ml) were mixed to 0.5 ml AgNPs solution containing 50 μg of Ag. On the 18th day, about 0.6 ml of the treatment solutions was injected *in ovo*, to each egg of treatment groups (except un-inj. Control) using a 24 gauge needle at the broader end of the egg, following the procedure described by Bhanja et al. (36).

### Housing and management

After hatch, all the chicks were vaccinated against Marek's disease, wing banded, and weighed. The chicks hatched from the different treatment groups were further distributed into two separate units (treatment-wise) where 50% of the chicks were vaccinated with Newcastle disease (ND) vaccine (F1 strain) on 4th d post-hatch and other 50% of the chicks (who were tested negative for antibody against ND, in serum) were kept unvaccinated. The ND-vaccinated birds were again vaccinated with inactivated ND vaccine strains on 24th d post-hatch. These two sets of chicks (vaccinated and unvaccinated) were housed in two different sheds but provided similar managemental practices. The unvaccinated unit was reared under utmost care to provide strict isolation and attended by a separate worker. The chicks were housed in 4-tier battery brooder cages. They were kept in a well-lit and ventilated open-sided house. The birds were provided with standard diets and management till 42 d of age. Food and water were provided *ad lib*.

### Performance parameters

Different pre-hatch parameters like pre-incubated egg weight, embryonic mortality, and percent hatchability were recorded. During post-hatch, body weight of individual birds and feed consumption by a group of eight birds (replicate wise) in each pen were recorded for 42 d to estimate the average daily gain (ADG), average daily feed intake (ADFI), and feed conversion ratio (FCR).

### Lymphoid weight and immune response

The relative development of the lymphoid organs was recorded at 42nd days post-hatch in vaccinated group. Six birds from each treatment (one bird from each replicate) were fasted for 8 h prior to being sacrificed. The weights of the bursa of Fabricius, thymus, and spleen were recorded and expressed as g kg−1 body weight. On 22nd d post-hatch, one ml of sheep RBC (1% v/v) suspension was injected intravenously to 12 birds from each treatment (two birds/replicate) from vaccinated and unvaccinated (data not presented) groups to study the primary antibody response against SRBC antigen. On 7 d post-immunization, 2 ml blood was collected from the wing vein and allowed to clot for serum separation. The antibody titer against SRBC was determined by hemagglutination (HA) method (37). The reciprocal of highest dilution showing clear agglutination was selected as end point of titer, and the value was expressed as log 2. *In vivo* response to PHA-P was evaluated by injecting about 0.1 ml PHA-P (1 mg/ml of PBS) intra-dermally into left foot web of 12 birds/treatment from the vaccinated and unvaccinated (data not presented) groups on 28th d post-hatch. Right foot web of the same birds received 0.1 ml sterile PBS and thus served as control. The skin thickness of foot webs (Right and Left) of the birds was measured by a micrometer at 0 and 24 h after injection of mitogen. Foot web index (FWI) was calculated by deducting the difference in thickness at 0 and 24 h of mitogen injected foot web with the difference in thickness of control foot web (38).

The specific antibody response against ND vaccine antigen was studied in the vaccinated group on 7th, 14^th^, and 21st d post-hatch. The hemagglutination inhibition (HI) test was performed in the serum. The plates were read under bright light. The highest dilution, at which there was complete inhibition of hemagglutination, was the HI titer value. Mercapto-ethanol-resistant antibodies (MER or IgG) against SRBC were determined as per the method described by Martin et al. (39) with slight modification. Mercapto-ethanol sensitive antibodies (MES or Ig M) against SRBC were calculated as the reduction from total SRBC HA titer due to 2-ME resistant titer.

### Newcastle disease virus challenge study

Eight birds each from vaccinated and unvaccinated groups of six treatments (a total of 96 birds) were selected based on their immune response to SRBC and PHA-P (one—low, two—moderate, and one—high responder bird from each treatment group) to eliminate individual variation for the challenge study. On 43rd d post-hatch, the birds were shifted to a challenge shed at IVRI, Izatnagar, India, to evaluate the response against virulent ND virus (vNDV). The Vndv-infected allantoic fluid was collected from Division of Pathology, IVRI, which was earlier recovered from a field outbreak. To get desired volume of virus, the infected fluid was propagated in 9th d old embryonated eggs as per the standard protocol (40). The presence of ND virus in the allantoic fluids was confirmed by typical lesions in the infected/dead embryos and HA tests. For calculating the required dose of NDV, a 10-fold serial dilution of the virus suspensions was carried out. The range of dilutions included at least two 10-fold dilutions above or below the dilution expected to contain the end point. At least five eggs were inoculated with each dilution. Separate needle and syringe for each dilution were used. The eggs were incubated for 4 days at 38°C. After 4 days, allantoic fluid was harvested from each egg and tested for the presence or absence of NDV. The application of the Reed and Muench mathematical technique was used to calculate the infectivity titer of the original suspension as 50 % embryo infectious or lethal dose (EID50/ELD50) as described below:


$$\begin{array}{l} \text{Index​}=\text{​ }\underline{(\%\text{ infected at dilution immediately above }50\%)-50\%}\\ \;(\%\text{ infected at dilution immediately above }50\%)-(\%\;\\ \text{ infected at dilution immediately below}\;50\%) \end{array}$$

### Recording of clinical signs and cumulative infection

Based on the EID50 /ELD50, eight birds from each treatment group were challenged with 0.1 ml (100 μl) of vNDV. Half of the dose was supplied as eye drop, and the remaining half was given intranasal, on the same side. The clinical symptoms and death time of the birds were recorded dpi (days postinoculation) and hour wise by the same observer six times a day. The extremely sick/terminally weak birds (showing severe neural symptoms with thick mucus in mouth) were euthanized and recorded as dead birds. A scoring pattern was followed as described by Oyebanji et al. (41) with little modifications. Based on the degree of sickness, the birds were classified into mild (Mi), moderate (Mo), severe(S), and dead/euthanized (D/E) categories on each dpi and granted with “clinical signs severity scores” of 10, 20, 30, and 40, respectively. The number of birds from each category was also expressed as percentage to the total number of live birds (*n* = L), at the end of previous dpi or in the beginning of that dpi. The “score obtained” was calculated as the sum of clinical signs severity scores of birds multiplied with number of birds in each category. Cumulative infection (CI), that is, the percentage of infection achieved until that dpi by the challenge virus, was estimated dpi wise and expressed as score obtained on that dpi / maximum possible score on that dpi × 100. The “maximum possible score” on that dpi was calculated as product of the clinical signs severity score for dead birds (40) and the total live birds on the beginning of that dpi. Post-mortem examination was carried out on dead chickens to determine the degree of disease severity. The vaccinated birds were killed on 2nd, 4th, and 6th dpi to observe the pathological changes.

### Expression of immune genes

Before and following the vNDV challenge, blood samples were collected at periodic intervals (0/8/24 h). PBMC cells were separated to study the expression pattern of IL2 and IL6 genes. Total mRNA from PBMC cells was isolated using TRIzol^®^ reagent following manufacturer's instructions. The purified RNA samples (using the SV Total RNA Isolation System, Promega Corporation, Madison, WI, USA) were quantified in a NanoDrop ND 1000 spectrophotometer (Thermo Fisher Scientific, Waltham, MA, USA). About 2 μg of total RNA was reverse-transcribed, after which real-time PCR was performed with complementary DNA and gene-specific primer pairs (8, 10) in an IQ5 cycler (BioRad, Hercules, CA, USA). The samples were first denatured for 5 min at 95 °C and then amplified using 45 cycles of 30 s at 95 °C for denaturation, 30 s at specific annealing temperature, and 30 s at 72 °C (elongation), followed by quantification. A melting curve was applied to verify the specificity of the product. For each complementary DNA, the reaction was performed in triplicate. The quantification of gene expression (fold change with respect to control) was done in REST 2009 software using the CT values obtained in Rt PCR study.

### Statistical analysis

Data were subjected to statistical analysis for one-way analysis of variance (ANOVA) using SPSS-16 software. Duncan's multiple range test was used for verifying significant difference among treatment means, and P < 0.05 was considered to be statistically significant.

## Results

### Incubational parameters and post-hatch growth

There was no difference (*P* ≥ 0.05) in the weight of fertile eggs used for *in ovo* injection, day old chick weight, chick weight to egg weight ratio, and percent hatchability (on fertile egg basis) between the treatment groups (Table 1). The 42nd d body weights, average daily feed intake (ADFI), average daily weight gain (ADG), and feed conversation ratio (FCR) during 0 to 42 d were also not different (*P* ≥ 0.05) between the treatment groups of vaccinated birds (Table 2). The performance of unvaccinated birds (data not presented) for 42nd d body weights, ADFI, ADG, and FCR ranged between 1,372.0 and 1,479 g, 69.3 and 73.5 g, 31.65 and 34.25 g, and 2.11 and 2.22, respectively.

**Table 1:** Effect of *in ovo* administration of silver nanoparticles (AgNPs) and critical nutrients on hatchability parameters of vaccinated birds.

**Groups^a^**	**Egg weight (g)**	**Chick weight (g)**	**Chick weight to egg weight ratio (%)**	**Hatchability on fertile egg set basis (%)**
Control	61.25	42.93	70.63	93.33
Sham control	60.28	43.30	71.96	91.67
AgNPs	60.53	43.13	71.25	90.20
AgNPs+AAs	60.10	44.05	73.37	90.91
AgNPs+Vits	60.90	43.93	72.16	93.85
AgNPs+TEs	60.4	44.28	73.33	92.86
SEM	0.36	0.29	0.46	ND
*P*-value	0.95	0.71	0.46	ND

a Control = un-injected, sham (sterile water), AgNPs = silver nanoparticles (50 μg), AgNPs+AAs = silver nanoparticles (50 μg) + amino acids (Methionine- 10 mg + Arginine-25 mg), AgNPs+Vits = silver nanoparticles (50 μg) + vitamins (Vit B1-72μg + Vit B6-140 μg) and AgNPs+TEs = silver nanoparticles (50 μg) + minerals (Zn-80 μg and Se-0.3 μg).

Values have been analyzed by one-way ANOVA. ND, not determined as single hatch was taken.

**Table 2:** Effect of *in ovo* administration of silver nanoparticles (AgNPs) and critical nutrients on performance of vaccinated birds.

**Groups^a^**	**42^nd^ d body weight (g)**	**ADFI, 0-42 d (g)**	**ADG, 0-42 d (g)**	**FCR, 0-42 d**
Control	1,392.9	71.84	32.12	2.23
Sham control	1,351.3	69.50	31.17	2.24
AgNPs	1,347.0	65.74	31.03	2.12
AgNps+AAs	1,366.4	70.50	31.48	2.24
AgNps+Vits	1,465.3	71.94	33.88	2.12
AgNps+TEs	1,443.1	70.63	33.32	2.12
SEM	20.2	1.00	0.48	0.03
*P*-value	0.459	0.638	0.443	0.659

a Control = un-injected, sham (sterile water), AgNPs = silver nanoparticles (50 μg), AgNPs+AAs = silver nanoparticles (50 μg) + amino acids (Methionine- 10 mg + Arginine-25 mg), AgNPs+Vits = silver nanoparticles (50 μg) + vitamins (Vit B1-72 μg + Vit B6-140 μg) and AgNPs+TEs = silver nanoparticles (50 μg) + minerals (Zn-80 μg and Se-0.3 μg).

Values have been analyzed by ANOVA.

### Lymphoid organ and immune response in vaccinated birds

The relative weight of bursa and thymus was similar in the treatment groups; however, the spleen weight was higher (*P* ≤ 0.05) in AgNPs+TEs group in comparison with control and sham control groups. Primary antibody titer against SRBC was similar between treatment groups of vaccinated birds; however, the foot web index was significantly higher in AgNPs+TEs group chicks than both the control groups (Table 3). Similarly, in the unvaccinated chicks (data not presented), HA titer against SRBC (log 2) and foot web index (mm) ranged from 8.0 to 9.5 and 0.39 to 0.60, respectively.

**Table 3:** Effect of *in ovo* administration of silver nanoparticles (AgNPs) and critical nutrients on the weight of immune organs in broiler chickens.

**Groups^a^**	**Bursa (g/100 g BW)**	**Spleen (g/100 g BW)**	**Thymus (g/100 g BW)**	**HA titer against SRBC (log 2)**	**Foot web index, mm)**
Control	0.132	0.171^a^	0.104	9.29	0.22^a^
Sham control	0.129	0.188^a^^b^	0.103	9.25	0.26^a^
AgNPs	0.131	0.203^b^^c^	0.108	9.43	0.41^a^^b^
AgNPs+AAs	0.133	0.195^a^^b^^c^	0.109	9.13	0.28^a^
AgNPs+Vits	0.131	0.203^b^^c^	0.099	9.63	0.34^a^^b^
AgNPs+TEs	0.132	0.223^c^	0.113	10.50	0.55^b^
SEM	0.003	0.005	0.003	0.29	0.03
*P*-value	1.000	0.020	0.911	0.805	0.049

a Control = un-injected, sham (sterile water), AgNPs = silver nanoparticles (50 μg), AgNPs+AAs = silver nanoparticles (50 μg) + amino acids (Methionine- 10 mg + Arginine-25 mg), AgNPs+Vits = silver nanoparticles (50 μg) + vitamins (Vit B1-72 μg + Vit B6-140 μg) and AgNPs+TEs = silver nanoparticles (50 μg) + minerals (Zn-80 μg and Se-0.3 μg).

Values have been analyzed by one-way ANOVA;

a, b, c Means bearing different alphabets in a column differ significantly (P < 0.05).

The 2-mercapto-ethanol-resistant (IgG) and sensitive (IgM) antibodies were similar (*P* ≥ 0.05) in treatment groups; however, apparently higher values of IgG and IGM were seen in AgNPs+TEs and AgNPs+AAs group chicks, respectively. The specific antibody response against ND vaccine was studied in the vaccinated group on 7th, 14th, and 21st d post-hatch. The HI titer values at all the periods were similar (*P* ≥ 0.05) between treatment groups; however, the AgNPs+Vits group chicks had apparently higher values at 7th and 14th d post-hatch (Table 4).

**Table 4:** Effect of *in ovo* administration of silver nanoparticles (AgNPs) and critical nutrients on the mercapto-ethanol-resistant (IgG) and sensitive (IgM) antibodies.

**Groups^a^**	**IgG**	**IgM**	**HI titer against ND vaccine (Log2)**		
**Control**	**2.71**	**6.57**	**Day 7**	**Day 14**	**Day 21**
			6.75	5.50	3.50
Sham control	3.00	6.14	6.25	5.25	3.25
AgNPs	2.86	6.00	6.75	5.75	4.00
AgNPs+AAs	2.43	7.43	6.25	5.00	2.50
AgNPs+Vits	3.29	7.00	7.75	6.75	3.25
AgNPs+TEs	3.43	6.00	6.25	5.50	2.75
SEM	0.17	0.27	0.22	0.25	0.17
P value	0.592	0.592	0.349	0.440	0.127

a Control = un-injected, sham (sterile water), AgNPs = silver nanoparticles (50 μg), AgNPs+AAs = silver nanoparticles (50 μg) + amino acids (Methionine- 10 mg + Arginine-25 mg), AgNPs+Vits = silver nanoparticles (50μg) + vitamins (Vit B1-72 μg + Vit B6-140 μg) and AgNPs+TEs = silver nanoparticles (50 μg) + minerals (Zn-80 μg and Se-0.3 μg).

Values have been analyzed by one-way ANOVA.

### Clinical signs, cumulative infection in VNDV challenged birds

Birds appeared clinically normal, and no visible symptoms were recorded on 1st dpi in all the treatment groups. On 2nd dpi, clinical signs such as ruffled feathers, depression, loss of appetite, and periorbital edema were observed. Five birds (62.5%), each from control, sham control, AgNPs+AAs, and AgNPs+Vits, showed mild infection on 2nd dpi, and the cumulative infection (CI) was estimated as 15.62% for these groups. However, in the AgNPs (37.5%) and AgNPs+TEs (50%) group, three or more birds were mildly sick with CI of 9.37 and 12.5%, respectively. By the end of 2nd dpi, two birds from each treatment group (vaccinated and unvaccinated) were euthanized to observe the gross pathological lesions. On 3rd dpi, the sick birds from different treatment groups (unvaccinated) progressed to complete depression, off fed with passage of greenish watery diarrhea, sternal recumbency, and head downing with nasal/lacrimal discharge. Three birds (50%), each from sham control, AgNPs, AgNPs+Vits, and AgNPs+TEs groups, and two birds (33.33%), from control and AgNPs+AAs groups, expressed severe clinical symptoms. Two unvaccinated birds (33.33%) each from control and sham control groups died by the end of 3rd dpi. Rest of the live birds from unvaccinated groups showed moderate degree of sickness (Table 5). The highest CI was observed in sham control (79.17%) and control (75%) groups, while it was the lowest in the AgNPs+AAs (58.33) until 3rd dpi. The CI was same (62.5%) in rest of the treatment groups (Table 6).

**Table 5:** Effect of *in ovo* administration of silver nanoparticles (AgNPs) and critical nutrients on classification of clinically ill birds on the basis of degree of severity (dpi wise) in treatment groups of unvaccinated birds.

**Groups^a^**	**2**^**nd**^ **dpi (*****n*** = **8)**					**3**^**rd**^ **dpi (*****n*** = **6)**					**4**^**th**^ **dpi (*****n*** = **L on 3rd dpi)**				**5**^**th**^ **dpi (*****n*** = **L on 4th dpi)**				**6t**^**h**^ **dpi (*****n*** = **L on 5th dpi)**	
	**No**	**Mi**	**Mo**	**E**	**L**	**Mi**	**Mo**	**S**	**D**	**L**	**Mo**	**S**	**D**	**L**	**Mo**	**S**	**D**	**L**	**D**	**L**
Control	3 (37.5)	5 (62.5)	0	2	6	0	2 (33.3)	2 (33.3)	2 (33.4)	4	0	2 (50)	2 (50)	2	0	0	2 (100)	0	0	0
Sham control	3 (37.5)	5 (62.5)	0	2	6	0	1 (16.7)	3 (50.0)	2 (33.3)	4	0	2 (50)	2 (50)	2	0	0	2 (100)	0	0	0
AgNPs	5 (62.5)	3 (37.5)	0	2	6	0	3 (50.0)	3 (50.0)	0	6	0	3 (50)	3 (50)	3	0	0	3 (100)	0	0	0
AgNPs+AAs	3 (37.5)	5 (62.5)	0	2	6	0	4 (66.7)	2 (33.3)	0	6	0	4 (66.6)	2 (33.3)	4	0	1 (25.0)	3 (75.0)	1	1 (100)	0
AgNPs+Vits	3 (37.5)	5 (62.5)	0	2	6	0	3 (50.0)	3 (50.0)	0	6	0	2 (33.3)	4 (66.7)	2	0	0	2 (100)	0	0	0
AgNPs+TEs	4 (50.0)	4 (50.0)	0	2	6	0	3 (50.0)	3 (50.0)	0	6	0	3 (50)	3 (50)	3	0	1 (33.3)	2 (66.7)	1	1 (100)	0

a Control = un-injected, sham (sterile water), AgNPs = silver nanoparticles (50 μg), AgNPs+AAs = silver nanoparticles (50 μg) + amino acids (Methionine- 10 mg + Arginine-25 mg), AgNPs+Vits = silver nanoparticles (50 μg) + vitamins (Vit B1-72 μg + Vit B6-140 μg) and AgNPs+TEs = silver nanoparticles (50 μg) + minerals (Zn-80 μg and Se-0.3 μg).

No, normal; Mi, mild; Mo, moderate; S, severe; D/E, dead/euthanized; L, remaining live birds at the end of that dpi; figures in the parenthesis are expressed as percentage to “n,” that is, live birds at the beginning of same dpi.

**Table 6:** Effect of *in ovo* administration of silver nanoparticles (AgNPs) and critical nutrients on scoring of clinically ill birds on the basis of degree of severity (dpi wise) from each treatment groups of unvaccinated birds.

	**2**^**nd**^ **dpi**			**3**^**rd**^ **dpi**			**4**^**th**^ **dpi**			**5**^**th**^ **dpi**			**6**^**th**^ **dpi**		
**Groups^a^**	**Score obtained**	**Max. possible score**	**C.I (%)**	**Score obtained**	**Max. possible score**	**C.I (%)**	**Score obtained**	**Max. possible score**	**C.I (%)**	**Score obtained**	**Max. possible score**	**C.I (%)**	**Score obtained**	**Max. possible score**	**C.I (%)**
Control	50	320	15.62	180	240	75.00	140	160	87.50	80	80	100	-	-	-
Sham control	50	320	15.62	190	240	79.17	140	160	87.50	80	80	100	-	-	-
AgNPs	30	320	9.37	150	240	62.50	210	240	87.50	120	120	100	-	-	-
AgNPs+AAs	50	320	15.62	140	240	58.33	200	240	83.33	150	160	93.75	40	40	100
AgNPs+Vits	50	320	15.62	150	240	62.50	220	240	91.67	80	80	100	-	-	-
AgNPs+TEs	40	320	12.50	150	240	62.50	210	240	87.50	110	120	91.67	40	40	100

a Control = un-injected, sham (sterile water), AgNPs = silver nanoparticles (50 μg), AgNPs+AAs = silver nanoparticles (50 μg) + amino acids (Methionine- 10 mg + Arginine-25 mg), AgNPs+Vits = silver nanoparticles (50 μg) + vitamins (Vit B1-72 μg + Vit B6-140 μg) and AgNPs+TEs = silver nanoparticles (50 μg) + minerals (Zn-80 μg and Se-0.3 μg).

Score obtained: the sum of clinical signs severity scores for mild (10), moderate (20), severe (30), and dead or euthanized (40) multiplied with number of birds in each category; maximum possible score: clinical signs severity score for dead birds (40) multiplied with the total live birds on the beginning of that dpi; CI: cumulative infection is the percentage of infection achieved on that dpi by the challenge virus as (score obtained/maximum possible score) × 100.

On 4th dpi, progressive symptoms in the forms of leg paralysis, muscle twitching, diarrhea, and anorexia preceded to severe symptoms of open mouth breathing, drooling salivation, total off feed, complete paralysis, and death. In the 4th dpi, two birds in control, sham control group (50%), and AgNPs+AAs group (33.3%), three birds (50.0%) each from of the AgNPs and AgNPs+TEs groups, and four birds from AgNPs+Vits group (66.6%) were found dead. Rest of the birds which survived exhibited complete inappetence and paralysis, and they were grouped under “severely sick” category (Table 5). The CI was the highest in the AgNPs+Vits (91.67%), whereas the AgNPs+AAs group maintained a lowest CI (83.33) until 4th dpi. The CI of 87.5% was found in control, sham control, AgNPs, and AgNPs+TEs groups (Table 6). However, two birds, one each from AgNPs+AAs and AgNPs+TEs groups, survived the entire 5th dpi and were dead on 6th dpi. Hence, the CI reached to the mark of 100% in most of the treatment groups, except AgNPs+AAs (93.75%) and AgNPs+TEs (91.67%) groups until 5th dpi. These two groups, particularly the AgNPs+AAs, exhibited maximum resistance to the NDV and attained 100% CI on 6th dpi (Table 6).

### Mean death time of challenged birds in unvaccinated group

**Figure 1:**
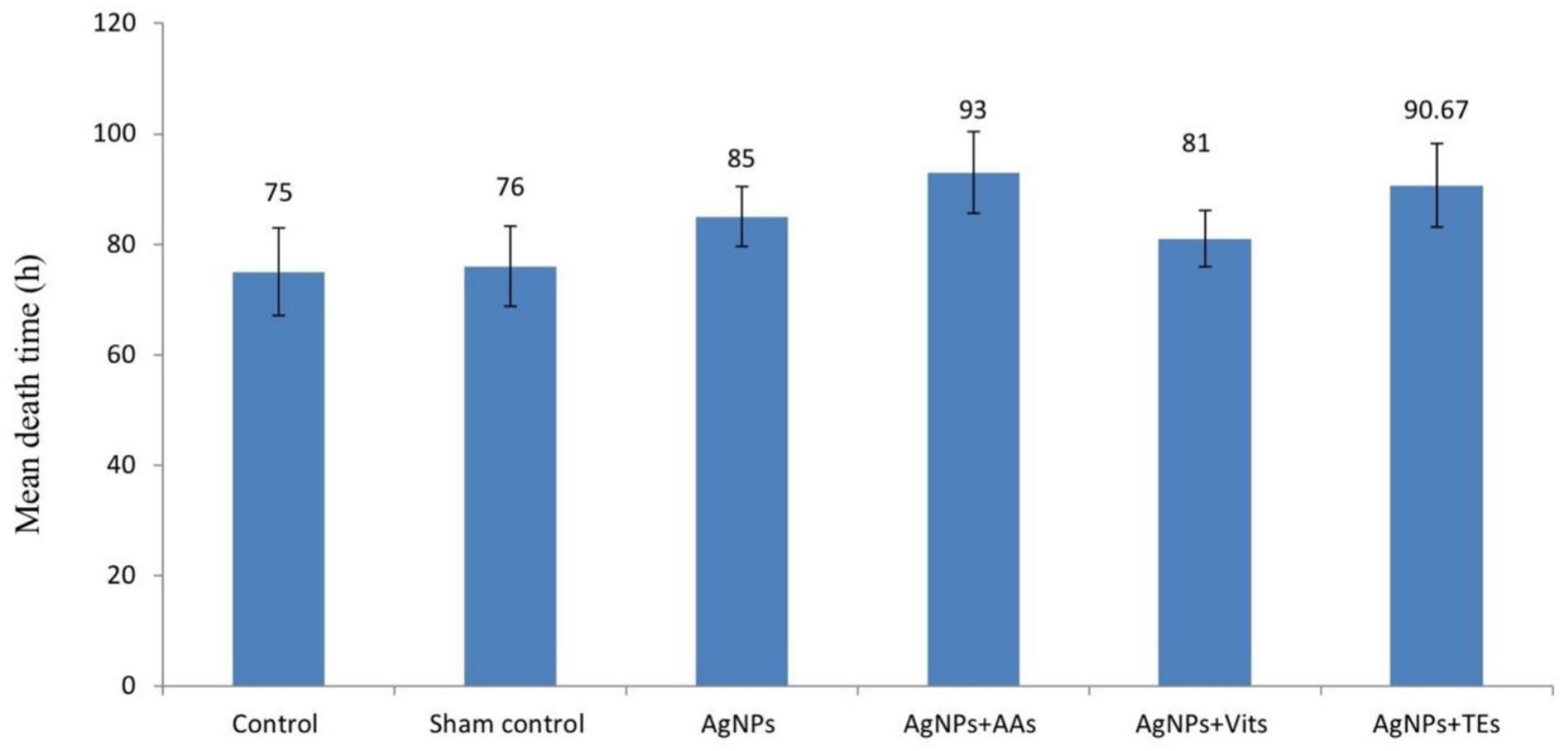
Effect of *in ovo* administration of silver nanoparticles (AgNPs) and critical nutrients on mean death time (h) of virulent ND virus (vNDV) challenged birds in unvaccinated group. Control = un-injected, sham (sterile water), AgNPs = silver nanoparticles (50 μg), AgNPs+AAs = silver nanoparticles (50 μg) + amino acids (Methionine- 10 mg + Arginine-25 mg), AgNPs+Vits = silver nanoparticles (50 μg) + vitamins (Vit B1-72 μg + Vit B6-140 μg) and AgNPs+TEs = silver nanoparticles (50 μg) + minerals (Zn-80 μg and Se-0.3 μg).

The estimated mean death time was apparently higher (*P* ≤ 0.01) in the birds of AgNPs+AAs and AgNPs+TEs groups than the controls and AgNPs+Vits (Figure 1).

### Gross lesions in unvaccinated group birds

The gross lesions in different visceral organs appeared in a similar chronology in the birds irrespective of treatment groups. However, there was a variation in the degree of severity, and the birds were categorized accordingly. Variation in the macro-pathological lesions of the trachea, bursa, brain, intestine, cecal tonsils, proventriculus, and kidney was examined and scored accordingly. The scores for intestinal lesions were awarded based on the observed lesions from outside (redden patches), lesions in lumen (hemorrhages with bloody content), and cecal tonsils (hemorrhages). A score of 30, 20, 10, or 0 was granted for severe (+++), moderate (++), mild (+), or absence (-) of typical ND lesions. The observed gross pathological lesions in different treatment groups are classified dpi wise and are presented in Table 7. The average scores for gross pathological lesions in different treatment groups have been presented in Table 8. The control group continued to show higher lesion scores (110) consistently up to 4th dpi, and the pathological lesions were less severe in the AgNPs+AAs (105) and AgNPs+TEs (93.33) group. Interestingly, the nutrient-injected groups showed comparatively less severe lesions consistently on each dpi as compared to control group chicks.

**Table 7:** Chronological appearance of gross lesions in unvaccinated challenged birds.

2^**nd**^ dpi	Congestion in the lower eye lid and periorbital swelling in few birds. Mild to moderate degree of hemorrhages in intestine. Mild hemorrhages in tracheal lumen in few birds.
3^**rd**^ dpi	Petechial hemorrhages and edema of lower eye lid, mild meningeal congestion, mottled spleen (multifocal and diffused white necrotic areas), mild to moderate edema and hemorrhages in intestine and cecal tonsils. Slight edematous bursa, congested kidneys and congested meningeal capillary was noticed.
4^**th**^ dpi	Moderate to severe hemorrhages in the cerebellum and brainstem region of skull, moderate to severe lesion in lymphoid organs and aggregates, watery greenish content in intestine, mild pinpoint hemorrhages at the tip of proventricular glands, moderate to severe mottling of spleen, multiple foci of edema, necrosis and hemorrhages in intestine and cecal tonsils, mild congestion in tracheal lumen, edematous bursa, congested kidneys and meningeal capillary congestion was noticed
5^**th**^ dpi	Mild lower eyelid congestion, several multifocal hemorrhages at the tip of the proventricular glands, enlarged whitish kidneys, severe congestion in meninges of brain, severe lesions in visceral organs, hemorrhagic edematous intestine with greenish/bloody intestinal content in lumen, severe hemorrhages in the cecal tonsil, severely mottled spleen and moderate to severe hemorrhages in tracheal lumen.
6^**th**^ dpi	Severe hemorrhages at cranial and caudal parts of tracheal lumen, semi-solid greenish content in intestine, severe multifocal pinpoint hemorrhages at the tip of the proventricular gland.

**Table 8:** Effect of ***in ovo*** administration of silver nanoparticles (AgNPs) and critical nutrients on the average scores of gross pathological lesions in different treatment groups (dpi wise).

**Groups^a^**	**2^nd^ dpi**	**3^rd^ dpi**	**4^th^ dpi**	**5^th^ dpi**	**6^th^ dpi**
Control	20 (*n* = 2)	45 (*n* = 2)	120 (*n* = 2)	160 (*n* = 2)	^*^ND
Sham control	15 (*n* = 2)	60 (*n* = 2)	110 (*n* = 2)	140 (*n* = 2)	^*^ND
AgNPs	15 (*n* = 2)	^*^ND	106.67 (*n* = 3)	146.67 (*n* = 3)	^*^ND
AgNPs+AAs	10 (*n* = 2)	^*^ND	105 (*n* = 2)	146.67 (*n* = 3)	140 (*n* = 1)
AgNPs+Vits	15 (*n* = 2)	^*^ND	110 (*n* = 4)	135 (*n* = 2)	^*^ND
AgNPs+TEs	15 (*n* = 2)	^*^ND	93.33 (*n* = 3)	140 (*n* = 2)	150 (*n* = 1)

a Control = un-injected, sham (sterile water), AgNPs = silver nanoparticles (50 μg), AgNPs+AAs = silver nanoparticles (50 μg) + amino acids (Methionine- 10 mg + Arginine-25 mg), AgNPs+Vits = silver nanoparticles (50 μg) + vitamins (Vit B1-72 μg + Vit B6-140 μg) and AgNPs+TEs = silver nanoparticles (50 μg) + minerals (Zn-80 μg and Se-0.3 μg).

* ND, not determined as no death or no birds were available from these groups, n is the number of birds necropsied on that dpi those contributed to the average scores for gross lesions on that dpi.

### Clinical signs and gross pathology in the vaccinated groups

Eight vaccinated birds from each treatment groups were challenged in the same manner as described for the unvaccinated birds. No clinical symptoms were observed in the vaccinated groups, except few birds with mild cases of ruffled feathers and greenish diarrhea. None of the vaccinated birds were dead until the end of the experiment (6th dpi). The birds were fully active, and there was no evidence of sickness or inappetence. Two birds were euthanized (treatment-wise) on 2nd and 4th dpi, and the remaining four birds were euthanized on 6th dpi, where only moderate levels of intestinal hemorrhages were observed.

### Relative expression of IL6 and IL-2 in the PBMC cells of unvaccinated challenged birds

**Figure 2:**
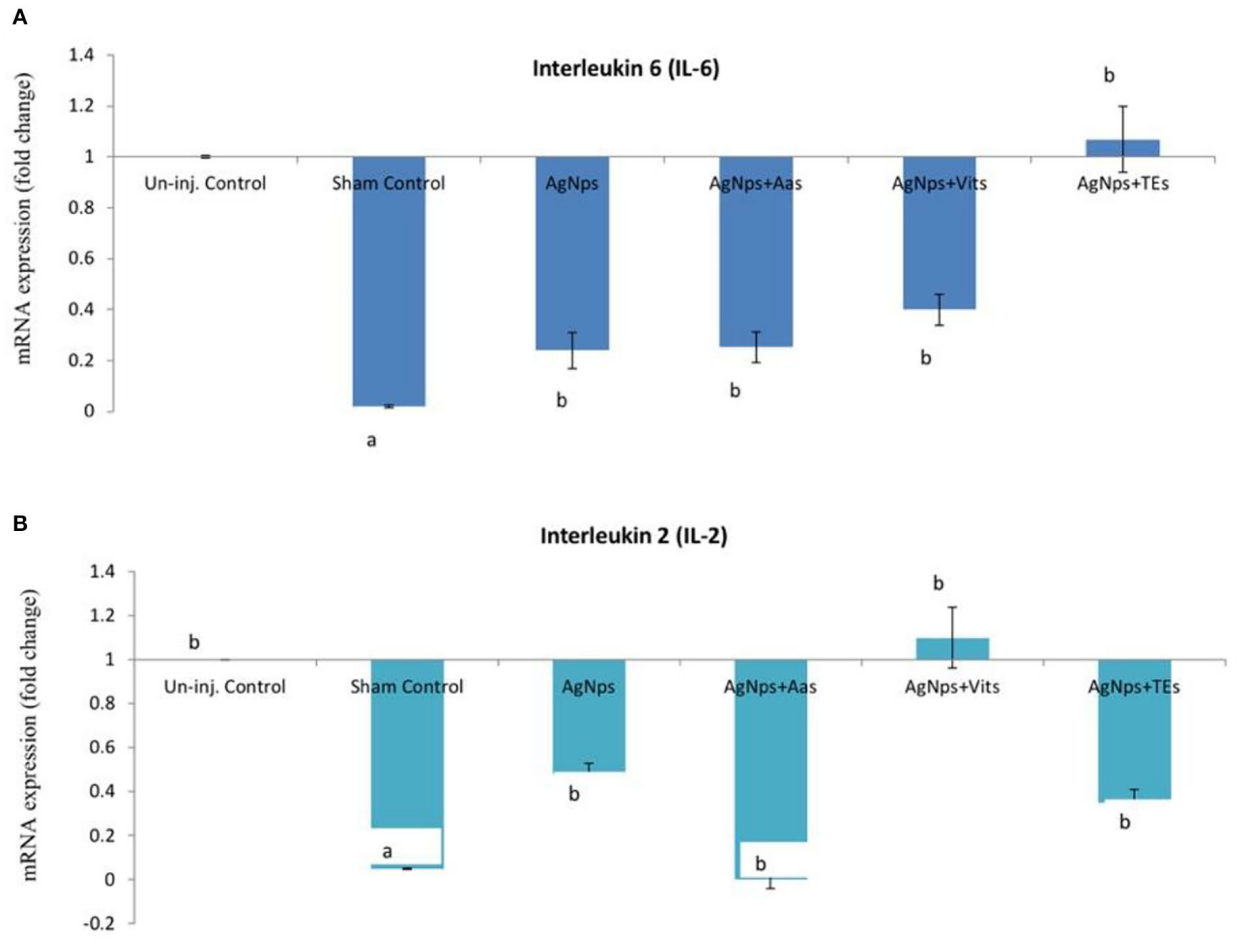
Effect of *in ovo* administration of silver nanoparticles (AgNPs) and critical nutrients on the relative expression of IL-6 **(A)** and IL-2 **(B)** genes before virulent ND virus (vNDV) challenge. Control = un-injected, sham (sterile water), AgNPs = silver nanoparticles (50 μg), AgNPs+AAs = silver nanoparticles (50 μg) + amino acids (Methionine- 10 mg + Arginine-25 mg), AgNPs+Vits = silver nanoparticles (50 μg) + vitamins (Vit B1-72 μg + Vit B6-140 μg) and AgNPs+TEs = silver nanoparticles (50 μg) + minerals (Zn-80 μg and Se-0.3 μg). ^a, b^Means bearing different alphabets in a histogram differ significantly (*P* < 0.05).

**Figure 3:**
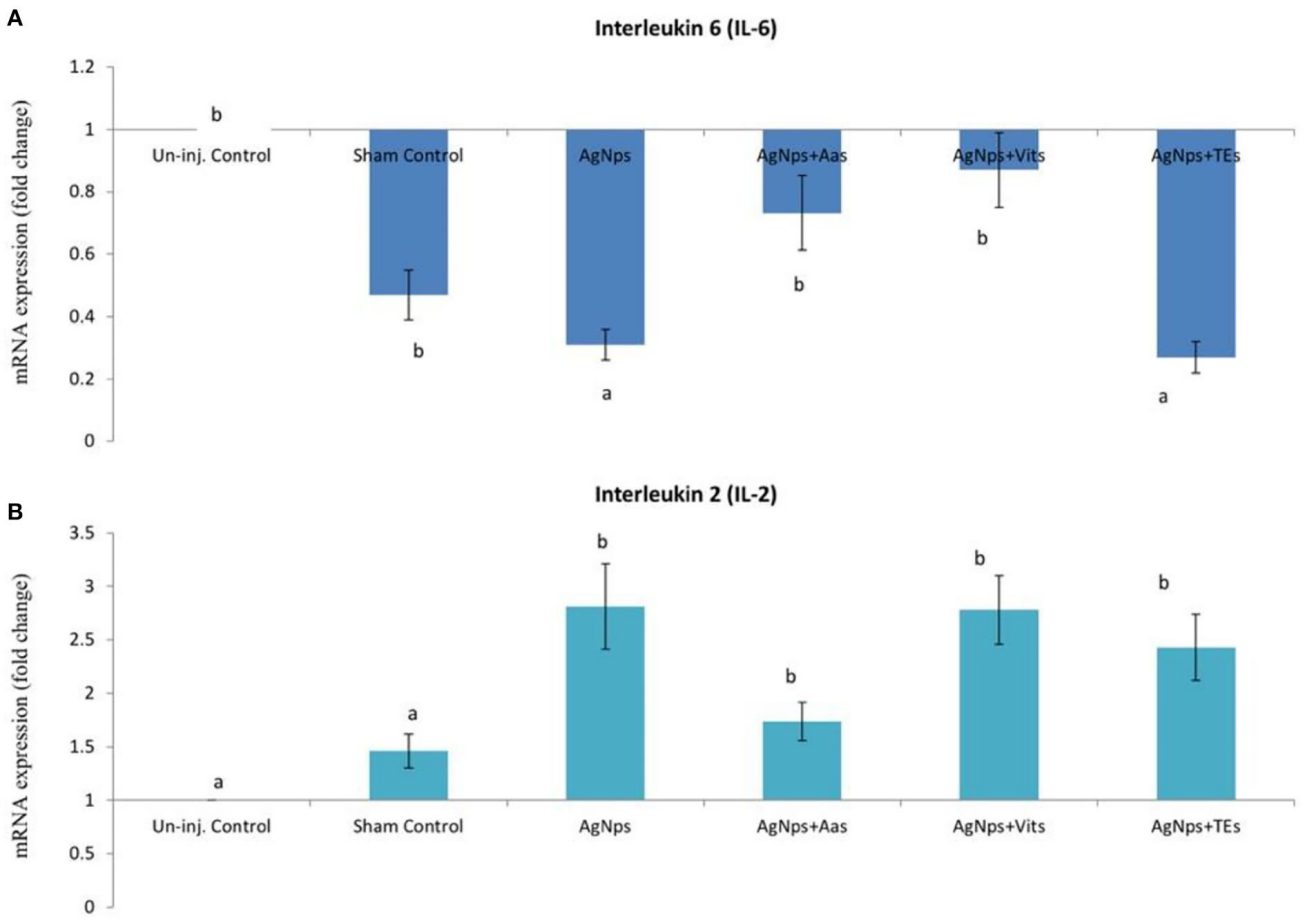
Effect of *in ovo* administration of silver nanoparticles (AgNPs) and critical nutrients on the relative expression of IL-6 **(A)** and IL-2 **(B)** genes 8 h post-virulent ND virus (vNDV) challenge. Control = un-injected, sham (sterile water), AgNPs = silver nanoparticles (50 μg), AgNPs+AAs = silver nanoparticles (50 μg) + amino acids (Methionine- 10 mg + Arginine-25 mg), AgNPs+Vits = silver nanoparticles (50 μg) + vitamins (Vit B1-72 μg + Vit B6-140 μg) and AgNPs+TEs = silver nanoparticles (50 μg) + minerals (Zn-80 μg and Se-0.3 μg). ^a, b^Means bearing different alphabets in a histogram differ significantly (*P* < 0.05).

**Figure 4:**
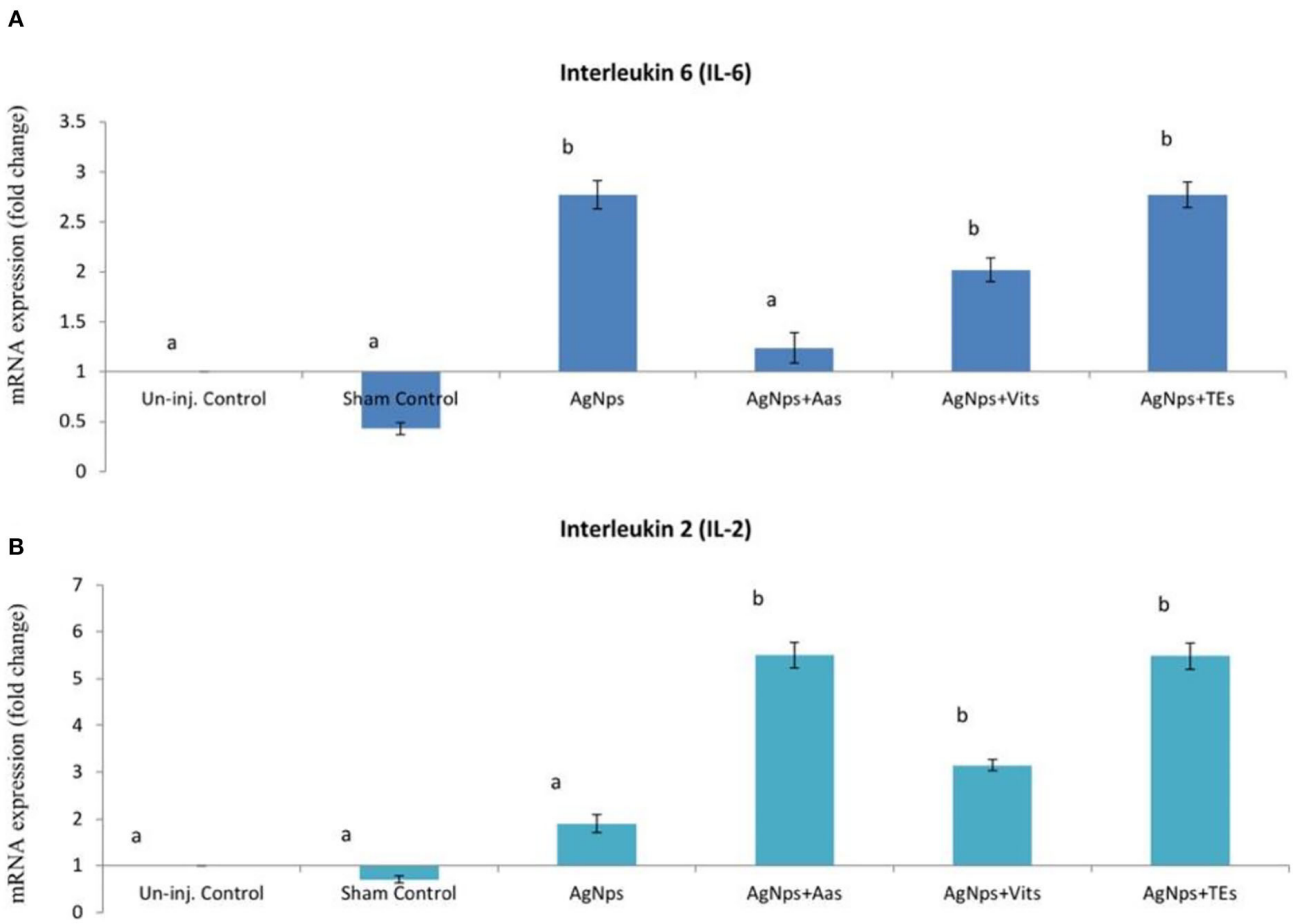
Effect of *in ovo* administration of silver nanoparticles (AgNPs) and critical nutrients on the relative expression of IL-6 **(A)** and IL-2 **(B)** genes 24 h post-virulent ND virus (vNDV) challenge. Control = un-injected, sham (sterile water), AgNPs = silver nanoparticles (50 μg), AgNPs+AAs = silver nanoparticles (50 μg) + amino acids (Methionine- 10 mg + Arginine-25 mg), AgNPs+Vits = silver nanoparticles (50 μg) + vitamins (Vit B1-72 μg + Vit B6-140 μg) and AgNPs+TEs = silver nanoparticles (50 μg) + minerals (Zn-80 μg and Se-0.3 μg). ^a, b^Means bearing different alphabets in a histogram differ significantly (*P* < 0.05).

There was no difference (*P* ≥ 0.05) in the relative expression of IL-6 or IL-2 genes just before vND virus challenge in AgNps treated groups; however, the expression significantly downregulated (*P* ≤ 0.05) in sham control group (Figures 2A,B). At 8 h post-vND virus challenge, IL-6 gene expression downregulated in AgNPs and AgNps+TEs group chicks as compared to un-injected chicks, but upregulation (*P* ≤ 0.05) of IL-2 gene was seen in all AgNPs group chicks, especially in AgNps+Vits and AgNps+TEs group (Figures 3A,B). Similarly, at 24 h post-vND virus challenge, relative expression of both the genes (IL-6 and IL-2) was significantly (*P* ≤ 0.05) upregulated in all AgNPs group chicks except that of IL-6 expression in AgNps+AAs group chicks (Figures 4A,B).

## Discussion

### Pre- and post-hatch performance in vaccinated birds

The chick weight increased to 2.6, 2.3, and 3.1% in the AgNPs+AAs, AgNPs+Vits, and AgNPs+TEs groups, respectively, in comparison with the control group. Similarly, the chick weight to egg weight ratio was higher (up to 3.87%) in the ***in ovo-***injected groups as compared to the un-injected control. The present observation is in consistent with earlier reports showing that there was no effect of AgNPs on incubational and hatching parameters like embryo development, hatchability, and relative chick weight (17, 42–44). Similarly, different nutrients when injected ***in ovo*** into the yolk sac or extra embryonic membranes had no adverse effects on embryo, with a little influence on hatchability (17, 45).

In the present study, ***in ovo*** supplementation of AgNPs+Vits (B1: 72μg and B6:140 μg) and AgNPs+TEs (Zinc 80 μg and selenium 0.3 μg) had 3.60–5.20 % higher 42nd d body weight as compared to un-injected control birds. Vitamin B1 plays a crucial role as a cofactor (thiamine pyrophosphate) in the conversion of glucose to energy, whereas vitamin B6, as pyridoxal phosphate, has role in amino acid transformations for protein synthesis. Altogether these two vitamins had also contributed for efficient feed conversion in the birds, helping them gaining more body weight. Goel et al. (25) observed that ***in ovo*** vitamin B1, B2, or E treated chicks attended higher body weight at 42nd d with a difference of 50 to 80g (3.6–5.8%) compared to un-injected control group. The effect of vitamin injection (B1, B2, and E) was more prominent in younger birds (till 21st d) with an increment of 5.3 to 13.3% body weight over control group (22). Role of trace elements, especially Zn as growth enhancer, cannot be ruled out. Goel et al. (27) reported higher growth-related gene expressions (i.e., Somatotropin, IGF-I, IGF-II, and mucin gene) in ***in ovo*** Zn injected chicks. Zn is found in more than 200 enzymes (Zn metalloenzymes) which perform catalytic or co-catalytic functions. Earlier reports have also shown that higher IGF-I gene expression correlated with early post-hatch growth rate and feed efficiency in broilers (46) and accelerated skeletal muscle development in quail embryos (47), though many researchers have recommended that AgNPs have an insignificant role in weight gain, feed intake, and FCR (8, 17, 19, 44, 48) but can act as carrier of critical nutrients (49). The hatchlings of today's improved broiler strains do not have an adequate energy store to meet the huge metabolic requirement and delay in transportation from hatchery to farms which further affects growth and immune system in these birds. ***In ovo*** feeding addresses these issues by providing nutrients to the young chicks through supplementation into the yolk sac or amnion of the developing embryo at later stage of incubation, thus complements the nutrient reserve of egg and post-hatch feed intake.

### Immune organ development and immune response

In this study, spleen weight was significantly higher (up to 30.4%) in AgNPs+TEs group, injected with Zn and Se in combination with AgNPs, in comparison with control and other groups. A significant increase in spleen weight (18.71%) was also observed in both AgNPs and AgNPs+Vits group as compared to the control group. Earlier reports have also recorded higher spleen weight in AgNPs treated birds (17, 50). Conversely, significant reduction in the lymphoid weight was also observed with increasing concentration of AgNPs in the diet (19, 50). In another study by Bakyaraj et al. (22), the thymus weight was significantly higher in the trace element-injected chicks than both amino acid and vitamins injected chicks. Goel et al. (27) also observed that the spleen weight was numerically higher in the Se supplemented chicks at 21st day post-hatch. The higher spleen weight in AgNPs injected chicks might be due to increase anabolic activity and subsequently stimulation of growth and development of lymphoid organs, as the structure of AgNPs enables to sustain atomic oxygen inside its octahedral holes for better oxygen supply (51).

In vaccinated birds, the *in vivo* immune response to PHA-P was significantly higher in the AgNPs+TEs group, in comparison with the control groups. This result correlates the previous studies where *in ovo* supplementation of AgNPs significantly enhanced the *in vivo* immune response to SRBC and PHA-P (17, 19, 27). Bhanja et al. (15) had also documented that the expression of cytokines (Th1 and Th2) and TLR genes, which are crucial for innate and adaptive immunity in chickens, was upregulated in AgNPs injected chicks. Goel et al. (27) reported significant upregulation of immune genes in Zn or Se injected chicks in comparison with the un-injected control chicks. AgNPs can increase activity of cell's immunity by stimulating heat shock protein (HSP) synthesis, without pro-inflammatory pathway activation as evidenced from lack of influence of AgNPs on expression of NF-kB, which is a transcriptional factor involved in defense of the organism (52). Furthermore, the antimicrobial and anti-inflammatory properties and possibility to enrich cells with oxygen improve immune system function of the organism supplemented with AgNPs. Selenium is considered as an immunological enhancing agent to enhance or recover immune functions of the organism (53). Earlier studies have also reported that Se played an important role in cellular immunity by increasing mitogen response to PHA-P (54) and wing web reaction in birds (55).

No significant difference was observed for the serum concentrations of IgG and IgM among the groups. However, the titer for IgG was higher in the AgNPs+TEs and that for IgM was higher in the AgNPs+AAs. Pineda et al. (42) found no discernible effects of pre-hatch and post-hatch silver nanoparticle exposure on plasma concentrations of IgG and IgM. Higher levels of IgM and IgG were produced by the birds under nano-silver treatment, but difference was insignificant (19). IgG is the major antibody isotype in chickens which mediate anaphylactic reactions, a function similar to IgE in mammals (56). In birds, the Fc region of IgG mediates most biological effector functions like complement fixation, anaphylactic reactions, and opsonization, whereas the Fab region contains the antigen-binding sites (57). The apparently higher IgG titer in the AgNPs+TEs can be correlated with the total Ab production against SRBC antigen but not against the ND vaccine.

### Response to VNDV challenge study

Eight birds from each treatment group (both vaccinated and unvaccinated) were infected with 10^7.4^ ELD_50_/0.1 ml/bird of ND challenge virus on 45th d post-hatch. Only the unvaccinated birds showed clinical symptoms and gross lesions. Most of the unvaccinated birds were found dead within 5th d postinfection (dpi) except two birds, one each from AgNPs+AAs and AgNPs+TEs, who died on 6th dpi. However, no death was observed in the vaccinated groups.

Our finding is in line with the study by Taylor et al. (58), where well-vaccinated chickens were challenged with high doses of vNDV daily for 10 days. Though all sham-vaccinated birds died by the fourth day postchallenge, no morbidity or mortality was reported in the NDV vaccinated birds even up to 14 d postchallenge. They also observed that repeated challenge with high-dose of vNDV did not overcome the vaccine immunity. In another experiment by Desingu et al. (59), the ND virus-infected chickens exhibited 100% mortality with marked lesions in proventriculus, intestine, spleen, and bursa. The cumulative infection percentage (dpi wise), mean death time (in h), and the average score for gross lesions show that the unvaccinated birds from AgNPs+AAs and AgNPs+TEs endured maximum resistance to the challenge virus. The mean death time was lowest for control birds and increased up to 13.0–24.0% in the *in ovo*-injected groups. This ensures the effect of combined supplementation of AgNPs and other nutrients in general, and the colloidal nano-silver in particular on immunocompetence and antiviral response in broiler chickens. Other studies, corroborating our findings, show that the amount and specificity of humoral antibodies induced by different vaccines affect viral replication and clinical protection (41, 58, 60). Antimicrobial and antiviral activity of silver nanoparticles (30–32) is mediated through Ag+ ions, which inhibit bacterial growth by suppressing respiratory enzymes and electron transport components which interfere with DNA functions (33) or through interaction with virus surface proteins (30) and viral genome (DNA or RNA).

### Differential expression of IL-6 and IL-2 gene

Significantly higher expression of IL-6 and IL-2 gene was seen in AgNPs and their combination with AAs, Vits, and TEs after vND virus challenge. Bhanja et al. (15) also reported enhanced expression of humoral (IL-6) and cell-mediated (IL-12) immunity gene in either AgNPs or Cys+AgNPs treated embryos following activation by LPS. In addition, the expression of humoral and cellular immunity-related genes was also increased in *in ovo* trace element (Zn and Se) and AgNPs supplemented chicks (17, 27). In chicken, T lymphocyte cells are important for immune system which after activation differentiates into T helper cells (61), which produce CD4 or CD8 antigens during embryogenesis (62). These cells either act as a source for pro-inflammatory cytokines (IL-6) that induces final maturation of B cells into antibody-secreting plasma cells (63) causing the proliferation and differentiation of immunoglobulin or by increasing T lymphocyte proliferation through production of IL-2 and involved in activation of NK natural killer and T cytolytic cells (64). Zinc is an essential component of thymulin, a hormone from thymus, and is involved in maturation and differentiation of T cells (65). In this study, AgNPs +AAs and AgNPs+ TEs group chicks after vND virus challenge had significantly higher expression of IL-6 and IL-2 expression and also had better response against SRBC and PHA-P.

## Conclusion

*In ovo* supplementation of AgNPs along with critical nutrients like amino acids, vitamins, and trace elements has shown promising result in improving post-hatch growth and immunity in broiler chickens, particularly that 50 μg/egg AgNPs in combination with vitamins (B1& B6) and trace elements (Zn & Se) improved the production performances and 50 μg/egg AgNPs with trace elements and amino acids enhanced immune response and resistance against vND virus challenge in broiler chickens. The antiviral property of AgNPs was apparently evident in our detailed challenge study. Nonetheless, the AgNPs to be used in therapeutic or prophylactic treatment in chickens, additional research is needed to determine the exact mode of antiviral mechanism and the highest safe dose in broilers, to augment the resistance to lethal avian viruses without creating new risk to humans or the environments.

## Data availability statement

The original contributions presented in the study are included in the article/supplementary material, further inquiries can be directed to the corresponding author.

## Ethics statement

All the experimental procedures on birds were carried out according to the recommendations and approval of the ICAR-Central Avian Research Institute, Izatnagar, India's Institute Animal Ethics Committee vides approval no. CARI/CPCSEA/2016/8 dated 23.08.2016 for the Purpose of Control and Supervision of Experiments on Animals in India.

## Author contributions

SB conceptualized the study, carried out biological study, analyzed data, and prepared the manuscript with input from AAlq, AAli, YA, VP, and AS. PR conducted the challenge experiment, data collection, and sample analysis. AG helped in sample collection, molecular, and gene expression study. MM helped in sample collection, sample analysis, and article writing. SD helped in molecular study, data analysis, and report writing. AAlq, AAli, YA, VP, and AS participated in the discussion and editing of the manuscript.

## Funding

This work was supported by Researchers Supporting Project number (RSP2022R439), King Saud University, Riyadh, Saudi Arabia.

## Conflict of interest

The authors declare that the research was conducted in the absence of any commercial or financial relationships that could be construed as a potential conflict of interest.

## Publisher's note

All claims expressed in this article are solely those of the authors and do not necessarily represent those of their affiliated organizations, or those of the publisher, the editors and the reviewers. Any product that may be evaluated in this article, or claim that may be made by its manufacturer, is not guaranteed or endorsed by the publisher.

## References

[1] Yalçin S Özkan S Türkmut L Siegel PB. Responses to heat stress in commercial and local broiler stocks. 2 Developmental stability of bilateral traits. Br Poult Sci. (2001) 42:153–60. 10.1080/00071660120048384 11421322

[2] Deeb N Shlosberg A Cahaner A. Genotype-by-environment interaction with broiler genotypes differing in growth rate. 4 Association between responses to heat stress and to cold-induced ascites. Poult Sci. (2002) 81:1454–62. 10.1093/ps/81.10.1454 12412909

[3] Shini S Kaiser P. Effects of stress, mimicked by administration of corticosterone in drinking water, on the expression of chicken cytokine and chemokine genes in lymphocytes. Stress. (2009) 12:388–99. 10.1080/10253890802526894 19006006

[4] Rajkumar U Reddy MR Rao S V Radhika K Shanmugam M. Evaluation of growth, carcass, immune response and stress parameters in naked neck chicken and their normal siblings under tropical winter and summer temperatures. Asian-Australasian J Anim Sci. (2011) 24:509–16. 10.5713/ajas.2011.10312

[5] Wilkinson KG Tee E Tomkins RB Hepworth G Premier R. Effect of heating and aging of poultry litter on the persistence of enteric bacteria. Poult Sci. (2011) 90:10–8. 10.3382/ps.2010-01023 21177438

[6] Abdel-Moneim A-ME Shehata AM Khidr RE Paswan VK Ibrahim NS El-Ghoul AA. Nutritional manipulation to combat heat stress in poultry – a comprehensive review. J Therm Biol. (2021) 98:102915. 10.1016/j.jtherbio.2021.102915 34016342

[7] Shehata AM Saadeldin IM Tukur HA Habashy WS. Modulation of heat-shock proteins mediates chicken cell survival against thermal stress. Animals. (2020) 10:2407. 10.3390/ani10122407 33339245 PMC7766623

[8] Sawosz E Grodzik M Zielińska M Niemiec T Olszańska B Chwalibog A. Nanoparticles of silver do not affect growth, development and DNA oxidative damage in chicken embryos. Arch für Geflügelkd. (2009) 73:208–13.

[9] Abdel-Moneim A-ME El-Saadony MT Shehata AM Saad AM Aldhumri SA Ouda SM. Antioxidant and antimicrobial activities of Spirulina platensis extracts and biogenic selenium nanoparticles against selected pathogenic bacteria and fungi. Saudi J Biol Sci. (2021) 29:1197–209. 10.1016/j.sjbs.2021.09.046 35197787 PMC8848030

[10] Abdel-Moneim A-ME Shehata AM Selim DA El-Saadony MT Mesalam NM Saleh AA. Spirulina platensis and biosynthesized selenium nanoparticles improve performance, antioxidant status, humoral immunity and dietary and ileal microbial populations of heat-stressed broilers. J Therm Biol. (2022) 104:103195. 10.1016/j.jtherbio.2022.103195 35180972

[11] Abdel-Moneim A-ME Shehata AM Mohamed NG Elbaz AM Ibrahim NS. Synergistic effect of Spirulina platensis and selenium nanoparticles on growth performance, serum metabolites, immune responses, and antioxidant capacity of heat-stressed broiler chickens. Biol Trace Elem Res. (2021) 200:68–779. 10.1007/s12011-021-02662-w 33674946

[12] Salem HM Ismael E Shaalan M. Evaluation of the effects of silver nanoparticles against experimentally induced necrotic enteritis in broiler chickens. Int J Nanomedicine. (2021) 16:6783. 10.2147/IJN.S319708 34675507 PMC8502061

[13] Dosoky WM Fouda MMG Alwan AB Abdelsalam NR Taha AE Ghareeb RY. Dietary supplementation of silver-silica nanoparticles promotes histological, immunological, ultrastructural, and performance parameters of broiler chickens. Sci Rep. (2021) 11:1–15. 10.1038/s41598-021-83753-5 33603060 PMC7892842

[14] Fouda MMG Dosoky WM Radwan NS Abdelsalam NR Taha AE Khafaga AF. Oral administration of silver nanoparticles–adorned starch as a growth promotor in poultry: Immunological and histopathological study. Int J Biol Macromol. (2021) 187:830–9. 10.1016/j.ijbiomac.2021.07.157 34331979

[15] Bhanja SK Hotowy A Mehra M Sawosz E Pineda L Vadalasetty KP. *In ovo* administration of silver nanoparticles and/or amino acids influence metabolism and immune gene expression in chicken embryos. Int J Mol Sci. (2015) 16:9484–503. 10.3390/ijms16059484 25923079 PMC4463600

[16] Hotowy A Sawosz E Pineda L Sawosz F Grodzik M Chwalibog A. Silver nanoparticles administered to chicken affect VEGFA and FGF2 gene expression in breast muscle and heart. Nanoscale Res Lett. (2012) 7:1–8. 10.1186/1556-276X-7-418 22827927 PMC3507702

[17] Goel A Bhanja SK Mehra M Majumdar S Mandal A. *In ovo* silver nanoparticle supplementation for improving the post-hatch immunity status of broiler chickens. Arch Anim Nutr. (2017) 71:384–94. 10.1080/1745039X.2017.1349637 28707480

[18] Erf GF Bottje WG Bersi TK Headrick MD Fritts CA. Effects of dietary vitamin E on the immune system in broilers: altered proportions of CD4 T cells in the thymus and spleen. Poult Sci. (1998) 77:529–37. 10.1093/ps/77.4.529 9565234

[19] Saki AA Salary J. The impact of *in ovo* injection of silver nanoparticles, thyme and savory extracts in broiler breeder eggs on growth performance, lymphoid-organ weights, and blood and immune parameters of broiler chicks. Poult Sci J. (2015) 3:165–72. 10.22069/psj.2015.2655

[20] Bhol KC Schechter PJ. Topical nanocrystalline silver cream suppresses inflammatory cytokines and induces apoptosis of inflammatory cells in a murine model of allergic contact dermatitis. Br J Dermatol. (2005) 152:1235–42. 10.1111/j.1365-2133.2005.06575.x 15948987

[21] Abdelsalam M Al-Homidan I Ebeid T Abou-Emera O Mostafa M Abd El-Razik M. Effect of silver nanoparticle administration on productive performance, blood parameters, antioxidative status, and silver residues in growing rabbits under hot climate. Animals. (2019) 9:845. 10.3390/ani9100845 31640236 PMC6826776

[22] Bakyaraj S Bhanja SK Majumdar S Dash BB. Post-hatch immunomodulation through *in ovo* supplemented nutrients in broiler chickens. J Sci Food Agric. (2012) 92:313–20. 10.1002/jsfa.4577 21800325

[23] Bhanja SK Mandal AB Agarwal SK Majumdar S. Modulation of post hatch-growth and immunocompetence through *in ovo* injection of limiting amino acids in broiler chickens. Indian J Anim Sci. (2012) 92:993–98.

[24] Bhanja SK Mandal AB. Effect of *in ovo* injection of critical amino acids on pre and post hatch growth, immunocompetence and development of digestive organs in broiler chickens. Asian-Australasian J Anim Sci. (2005) 18:524–31. 10.5713/ajas.2005.524

[25] Goel A Pande V Bhanja SK Mehra M Majumdar S. Effects of *in ovo* administration of vitamins on post hatch-performance, immunocompetence and blood biochemical profiles of broiler chickens. Indian J Anim Sci. (2013) 83:916–21. Available online at: https://epubs.icar.org.in/index.php/IJAnS/article/view/33025

[26] Saleh AA Ebeid TA. Feeding sodium selenite and nano-selenium stimulates growth and oxidation resistance in broilers. South African J Anim Sci. (2019) 49:176–84. 10.4314/sajas.v49i1.20

[27] Goel A Bhanja SK Mehra M Mandal AB Pande V. *In ovo* trace element supplementation enhances expression of growth genes in embryo and immune genes in post-hatch broiler chickens. J Sci Food Agric. (2016) 96:2737–45. 10.1002/jsfa.7438 26399199

[28] El-Deep MH Ijiri D Ebeid TA Ohtsuka A. Effects of dietary nano-selenium supplementation on growth performance, antioxidative status, and immunity in broiler chickens under thermoneutral and high ambient temperature conditions. J Poult Sci. (2016) 53:274–83. 10.2141/jpsa.0150133 32908394 PMC7477162

[29] Sagar D Nasir AM Mandal AB Kapil D Jubeda B Tyagi PK. Comparative study on the responses of broiler chicken to hot and humid environment supplemented with different dietary levels and sources of selenium. J Therm Biol. (2020) 88:102515. 10.1016/j.jtherbio.2020.102515 32125992

[30] Galdiero S Falanga A Vitiello M Cantisani M Marra V Galdiero M. Silver nanoparticles as potential antiviral agents. Molecules. (2011) 16:8894–918. 10.3390/molecules16108894 22024958 PMC6264685

[31] Lara HH Ayala-Nuñez N V Ixtepan-Turrent L Rodriguez-Padilla C. Mode of antiviral action of silver nanoparticles against HIV-1. J Nanobiotechnology. (2010) 8:1–10. 10.1186/1477-3155-8-1 20145735 PMC2818642

[32] Sun RW-Y Chen R Chung NP-Y Ho C-M Lin C-LS Che C-M. Silver nanoparticles fabricated in Hepes buffer exhibit cytoprotective activities toward HIV-1 infected cells. Chem Commun. (2005) 5059–5061. 10.1039/b510984a 16220170

[33] Li M Mizuuchi M Burke TR Jr Craigie R. Retroviral DNA integration: reaction pathway and critical intermediates. EMBO J. (2006) 25:1295–304. 10.1038/sj.emboj.7601005 16482214 PMC1422164

[34] Shehata AM Paswan VK Attia YA Abdel-Moneim A-ME Abougabal MS Sharaf M. Managing gut microbiota through *in ovo* nutrition influences early-life programming in broiler chickens. Animals. (2021) 11:3491. 10.3390/ani11123491 34944266 PMC8698130

[35] NRC. Nutrient Requirements of Poultry: 1994. Washington, DC: National Academies Press (1994).

[36] Bhanja SK Mandal AB Johri TS. Standardization of injection site, needle length, embryonic age and concentration of amino acids for *in ovo* injection in broiler breeder eggs. Indian J Poult Sci. (2004) 39:105–11.

[37] Van der Zijpp AJ. The effect of genetic origin, source of antigen, and dose of antigen on the immune response of cockerels. Poult Sci. (1983) 62:205–11. 10.3382/ps.0620205 6835897

[38] Corrier DE DeLoach JR. Evaluation of cell-mediated, cutaneous basophil hypersensitivity in young chickens by an interdigital skin test. Poult Sci. (1990) 69:403–8. 10.3382/ps.0690403 2345722

[39] Martin A Gross WB Siegel PB. IgG and IgM responses in high and low antibody-selected lines of chickens. J Hered. (1989) 80:249–52. 10.1093/oxfordjournals.jhered.a110844 2732455

[40] Brucellosis OIEB. Manual of Diagnostic Tests and Vaccine for Terrestrial Animal's. 5th ed. Paris: Off Intern des Epiz Paris (2008). p. 242–62.

[41] Oyebanji VO Emikpe BO Oladele OA Osowole OI Salaam A Odeniyi MA. Clinicopathological evaluation of Newcastle disease virus vaccination using gums from Cedrela odorata and Khaya senegalensis as delivery agents in challenged chickens. Int J Vet Sci Med. (2017) 5:135–42. 10.1016/j.ijvsm.2017.09.002 30255062 PMC6137849

[42] Pineda L Chwalibog A Sawosz E Lauridsen C Engberg R Elnif J. Effect of silver nanoparticles on growth performance, metabolism and microbial profile of broiler chickens. Arch Anim Nutr. (2012) 66:416–29. 10.1080/1745039X.2012.710081 22889095

[43] Pineda L Sawosz E Hotowy A Elnif J Sawosz F Ali A. Effect of nanoparticles of silver and gold on metabolic rate and development of broiler and layer embryos. Comp Biochem Physiol Part A Mol Integr Physiol. (2012) 161:315–9. 10.1016/j.cbpa.2011.11.013 22182492

[44] Grodzik M Sawosz E. The influence of silver nanoparticles on chick embryo development and bursa of Fabricius morphology. J Anim Feed Sci. (2006) 15:111. 10.22358/jafs/70155/2006

[45] Ohta Y Yoshida T Tsushima N Kidd MT. The needle bore diameter for *in ovo* amino acid injection has no effect on hatching performance in broiler breeder eggs. J Poult Sci. (2002) 39:194–7. 10.2141/jpsa.39.194

[46] Kocamis H Yeni YN Kirkpatrick-Keller DC Killefer J. Postnatal growth of broilers in response to *in ovo* administration of chicken growth hormone. Poult Sci. (1999) 78:1219–26. 10.1093/ps/78.8.1219 10472850

[47] Deprem T Guelmez N. The effects of *in ovo* insulin-like growth factor-1 on embryonic development of musculus longus colli dorsalis in Japanese quail. Turkish J Vet Anim Sci. (2007) 31:233–40. Available online at: https://journals.tubitak.gov.tr/veterinary/vol31/iss4/4

[48] Ahmadi F Kurdestany AH. The impact of silver nano particles on growth performance, lymphoid organs and oxidative stress indicators in broiler chicks. Glob Vet. (2010) 5:366–70.

[49] Bhanja SK Goel A Pandey N Mehra M Majumdar S Mandal AB. *In ovo* carbohydrate supplementation modulates growth and immunity-related genes in broiler chickens. J Anim Physiol Anim Nutr. (2015) 99:163–73. 10.1111/jpn.12193 24797673

[50] Andi MA Hashemi M Ahmadi F. Effects of feed type with/without nanosil on cumulative performance, relative organ weight and some blood parameters of broilers. Glob Vet. (2011) 7:605–9.

[51] Studnicka A Sawosz E Grodzik M Chwalibog A Balcerak M. Influence of nanoparticles of silver/palladium alloy on chicken embryos' development. Anim Sci. (2009) 46:237–42.

[52] Sawosz E Chwalibog A Szeliga J Sawosz F Grodzik M Rupiewicz M. Visualization of gold and platinum nanoparticles interacting with *Salmonella enteritidis* and Listeria monocytogenes. Int J Nanomedicine. (2010) 5:631. 10.2147/IJN.S12361 20856838 PMC2939708

[53] Ru-duan W Chang-sen W Zuo-hua F Yi L. Investigation on the effect of selenium on T lymphocyte proliferation and its mechanisms. J Tongji Med Univ. (1992) 12:33–8. 10.1007/BF02887756 1619693

[54] Biswas A Mohan J Sastry KVH. Effect of higher levels of dietary selenium on production performance and immune responses in growing Japanese quail. Br Poult Sci. (2006) 47:511–5. 10.1080/00071660600830629 16905478

[55] Kiremidjian-Schumacher L Roy M. Selenium and immune function. Z Ernahrungswiss. (1998) 37:50–6. 9558729

[56] Carlander D. Avian IgY antibody: *in vitro* and *in vivo*. Acta Universitatis Upsaliensis. (2002) p. 53.

[57] Härtle S Magor KE Göbel TW Davison F Kaspers B. Structure and evolution of avian immunoglobulins. In: Avian Immunology. Academic Press (2022). p. 101–19. 10.1016/B978-0-12-818708-1.00023-3

[58] Taylor TL Miller PJ Olivier TL Montiel E Cardenas Garcia S Dimitrov KM. Repeated challenge with virulent Newcastle disease virus does not decrease the efficacy of vaccines. Avian Dis. (2017) 61:245–9. 10.1637/11555-120816-ResNote.1 28665733

[59] Desingu PA Singh SD Dhama K Kumar ORV Malik YS Singh R. Clinicopathological characterization of experimental infection in chickens with sub-genotype VIIi Newcastle disease virus isolated from peafowl. Microb Pathog. (2017) 105:8–12. 10.1016/j.micpath.2017.01.057 28163156

[60] Miller PJ Afonso CL El Attrache J Dorsey KM Courtney SC Guo Z. Effects of Newcastle disease virus vaccine antibodies on the shedding and transmission of challenge viruses. Dev Comp Immunol. (2013) 41:505–13. 10.1016/j.dci.2013.06.007 23796788

[61] Spellberg B Edwards JE Jr. Type 1/Type 2 immunity in infectious diseases. Clin Infect Dis. (2001) 32:76–102. 10.1086/317537 11118387

[62] Lillehoj HS Trout JM. Avian gut-associated lymphoid tissues and intestinal immune responses to Eimeria parasites. Clin Microbiol Rev. (1996) 9:349–60. 10.1128/CMR.9.3.349 8809465 PMC172898

[63] Jones SA. Directing transition from innate to acquired immunity: defining a role for IL-6. J Immunol. (2005) 175:3463–8. 10.4049/jimmunol.175.6.3463 16148087

[64] Cantrell DA Collins MK Crumpton MJ. Autocrine regulation of T-lymphocyte proliferation: differential induction of IL-2 and IL-2 receptor. Immunology. (1988) 65:343. 3264805 PMC1385470

[65] Prasad AS. Zinc: role in immunity, oxidative stress and chronic inflammation. Curr Opin Clin Nutr Metab Care. (2009) 12:646–52. 10.1097/MCO.0b013e3283312956 19710611

